# Web-Based Protein Interactions Calculator Identifies Likely Proteome Coevolution with Alzheimer’s Disease-Associated Proteins

**DOI:** 10.3390/genes13081346

**Published:** 2022-07-27

**Authors:** Katrisa M. Ward, Brandon D. Pickett, Mark T. W. Ebbert, John S. K. Kauwe, Justin B. Miller

**Affiliations:** 1 Department of Biology, Brigham Young University, Provo, UT 84602, USA; katrisa14@gmail.com (K.M.W.); pickettbd@byu.edu (B.D.P.); kauwe@byu.edu (J.S.K.K.); 2 Sanders-Brown Center on Aging, University of Kentucky, Lexington, KY 40536, USA; mark.ebbert@uky.edu; 3 Division of Biomedical Informatics, Department of Internal Medicine, University of Kentucky, Lexington, KY 40506, USA; 4 Department of Neuroscience, University of Kentucky, Lexington, KY 40506, USA; 5 Department of Pathology and Laboratory Medicine, University of Kentucky, Lexington, KY 40506, USA

**Keywords:** Alzheimer’s disease, mutual information, protein interactions, protein interactions calculator, coevolution, proteomics

## Abstract

Protein–protein functional interactions arise from either transitory or permanent biomolecular associations and often lead to the coevolution of the interacting residues. Although mutual information has traditionally been used to identify coevolving residues within the same protein, its application between coevolving proteins remains largely uncharacterized. Therefore, we developed the Protein Interactions Calculator (PIC) to efficiently identify coevolving residues between two protein sequences using mutual information. We verified the algorithm using 2102 known human protein interactions and 233 known bacterial protein interactions, with a respective 1975 and 252 non-interacting protein controls. The average PIC score for known human protein interactions was 4.5 times higher than non-interacting proteins (*p* = 1.03 × 10^−108^) and 1.94 times higher in bacteria (*p* = 1.22 × 10^−35^). We then used the PIC scores to determine the probability that two proteins interact. Using those probabilities, we paired 37 Alzheimer’s disease-associated proteins with 8608 other proteins and determined the likelihood that each pair interacts, which we report through a web interface. The PIC had significantly higher sensitivity and residue-specific resolution not available in other algorithms. Therefore, we propose that the PIC can be used to prioritize potential protein interactions, which can lead to a better understanding of biological processes and additional therapeutic targets belonging to protein interaction groups.

## 1. Introduction

Protein–protein interaction networks can help researchers understand cellular function, predict genetic variant effects, and map basic biological processes [1,2,3]. These networks have direct disease implications. Many genetic variants in complex diseases, including Alzheimer’s disease, are known to affect the same molecular pathways and are likely to have a similar functional impact [4,5]. Although several protein interaction databases exist [1,6,7,8,9,10,11,12,13,14,15,16,17,18,19], they generally lack a residue-specific resolution and the ease of use that may aid in their wider adaptation. Furthermore, interacting proteins are not necessarily similar to each other, nor do similar proteins necessarily interact, which is the underlying assumption of most computationally-based prediction algorithms [2]. An evolutionary approach offers a higher resolution to identify amino acid residue coevolution that can be used to identify interacting proteins.

Direct coupling analysis models built using the maximum entropy principle have recently identified a three-dimensional structure and conformational effects caused by mutations in protein sequences [20,21,22,23,24,25,26,27,28]. While this modeling generally performs well on structure prediction, it underperforms compared to a simpler mutual information maximization in predicting residue-specific interactions between protein families [29]. Mutual information is an element of information theory that measures the dependence of any two variables [30]. It may be used as a numerical measurement of coevolution between two amino acid residues when applied to multiple sequence alignments spanning various species [31,32,33,34] since functionally-conserved mutations often become fixed in a population and are evolutionarily conserved [35]. Coevolution often results from residue contact pairing or protein folding that indicates a protein–protein interaction [36,37,38] and has been used in various capacities to detect phosphate signaling mechanisms between proteins in *Escherichia coli* [39] and pre-screen potential interactions between different DNA-replicating proteins in *Arabidopsis thaliana* [40]. Until recently, however, computational complexity and limited data availability have made it difficult to perform these types of analyses [41,42,43]. Therefore, available mutual information calculators have limited utility in predicting protein–protein interactions because their results often lack cross-study normalization, require excessive computational resources, have limited documentation, or exclusively detect relationships between positions within a single gene or protein [31,32,33,34].

Here, we present the Protein Interaction Calculator (PIC), which is optimized for detecting residue interactions between protein sequences. This calculator is not meant for the prediction of protein folding but for the prediction of the interactions between distinct proteins. The results from the PIC are normalized to allow direct comparisons between different analyses, even when the underlying protein sequences are vastly different. Additionally, the PIC provides a probability that the two proteins interact based on extensive validation using known interactions in both humans and bacteria. We performed pairwise comparisons of all Alzheimer’s disease-associated proteins against all human proteins to determine the extent to which coevolution might confound Alzheimer’s disease research. We identified 1933 protein interactions that are very likely to occur (>95% probability) and have displayed those interactions on our web-based application (https://pic.byu.edu). The website also allows users to upload two multiple sequence alignments and calculate the probability that the residues within those sequences will interact. A command line interface (https://github.com/kauwelab/pic) is also available for users to locally perform multiple queries in tandem. We anticipate that the PIC will be used to screen for protein interactions to identify potential secondary effects of genetic variants implicated in disease studies.

## 2. Materials and Methods

### 2.1. Mutual Information

The PIC uses mutual information to find relationships between proteins by comparing each residue position on one protein to each residue position on another protein. Two multiple sequence alignments are required as input: one for each protein. The two files must contain at least 100 species in common (i.e., the same header lines, although not necessarily in the same order) to ensure sufficient sampling of the protein and its residues. All of the species not found in both files will be dropped from the analyses. Because orthologs in multiple-sequence alignments can contain insertions and deletions, a user-defined reference species must be used to report the residue positions in the final output file. The missing positions in the multiple sequence alignment (i.e., “-”) in the reference species are dropped from the analyses. A mutual information score is calculated for each residue–residue pairing, and the highest residue–residue score is used to determine whether a possible relationship exists between the two proteins.

(1) $$MI\left(X,Y\right)=\underset{y\in Y}{\sum }\underset{x\in X}{\sum }p_{XY}\left(x,y\right)\times log\left(\frac{p_{XY}\left(x,y\right)}{p_{X}\left(x\right)p_{Y}\left(y\right)}\right)$$

Equation (1) shows the base equation for calculating mutual information (MI), where X is the set of residues occurring at a given position on protein 1, and Y is the set of residues occurring at a given position on protein 2. The above equation is a summation of several partial mutual information scores, one for each possible combination of residues from X and Y. $p_{XY}\left(x,y\right)\;$is the proportion of times that an individual combination occurs in a species (i.e., the set of species where x occurs at the position of interest on protein 1 is the same set of species in which y occurs at the position of interest on protein 2). $p_{X}\left(x\right)$ and $p_{Y}\left(y\right)$ are defined as the proportions of x and y relative to X and Y, respectively [30]. In other words, $p_{X}\left(x\right)$ indicates how often a specific residue occurs at a given position on protein 1, and $p_{Y}\left(y\right)$ indicates how often a specific residue occurs at a given position on protein 2. Intra-protein partial mutual information scores are not included in the calculation.

### 2.2. Filters

We optimized the calculations by determining a minimum threshold for $p_{XY}\left(x,y\right)$, $p_{X}\left(x\right)$, and $p_{Y}\left(y\right)$. Since $p_{X}\left(x\right)$ and $p_{Y}\left(y\right)$ are the same type of value arising from two separate but similar sets, the filter is the same for both values and is referred to as the $p_{X}\left(x\right)$ threshold. The minimum $p_{X}\left(x\right)$ threshold is set as a fixed value, whereas the minimum $p_{XY}\left(x,y\right)$ threshold is based on $p_{X}\left(x\right)$. In theory, if events x and y are fully independent (i.e., no coevolution), $p_{XY}\left(x,y\right)$ would equal $p_{X}\left(x\right)$ × $p_{Y}\left(y\right)$, resulting in a partial mutual information score for that residue combination of zero, which would not contribute to the overall score for the pairing. However, we found statistical artifacts in $p_{XY}\left(x,y\right)$ values resulting in slightly nonrandom results contributing to $MI\left(X,Y\right)$, which ultimately decreased the accuracy of the mutual information scores. Therefore, we decided to establish a filter for $p_{XY}\left(x,y\right)$ to ensure that the probability of the partial mutual information scores is above random. Additional details on how these filters were established are available in Text S1.

In addition to the partial mutual information score filters, each residue–residue score was divided by the average score for the entire protein–protein comparison. This method is similar to the average product correction, which successfully reduces background noise and increases accuracy in other mutual information-based protein analyses [42]. The scores are then multiplied by 10,000 for improved readability since normalized scores are generally less than 0.00001.

Three outputs are possible: (1) a summary file in tab-separated value (TSV) format, (2) a detailed file with position-wise scores in comma-separated value (CSV) format, and (3) a heatmap image. The summary file is better suited for studying multiple protein interactions because the output simply appends a single line to the file containing the highest score, positions of interaction for the highest score, the likelihood of interaction based on the highest score, and the names of the two proteins. The same output file name may be used for different analyses because the program appends and does not overwrite, resulting in a neat file summarizing several results of the algorithm. Since the detailed file provides each position-wise score, it is better suited to single protein–protein analyses. Any combination of the outputs may be generated when run on the command-line. On the website, the detailed (CSV) file and heatmap are made available for the user to download, while an output, such as the summary (TSV) file (i.e., the MI score and the likelihood of that score being a true interaction) is reported on the screen. The likelihood is determined based on the distributions of several comparisons of known and unknown protein interactions discussed below.

### 2.3. Verification I

We first needed to generate multiple sequence alignments of orthologs spanning at least 100 species to validate our algorithm. All sequence data were downloaded from RefSeq [44,45,46] in December 2019, and multiple sequence alignments were generated using default parameters in Clustal Omega [47]. The longest isoform of each protein was chosen as representative of that protein for each species. We identified 15,092 proteins that were annotated in at least 100 species, which were used to generate multiple sequence alignments that could be used for these algorithmic validations.

Next, we generated two separate lists of protein pairs that (1) were known to interact and (2) had no known interactions. In December 2020, we downloaded protein interaction networks from GPS-Prot [17], which is a database of protein interaction networks that includes indirect interactions where the proteins’ interactions may be several degrees removed from the primary interaction. Tables S1 and S2 list the interactions used for validation.

We chose a random representative sample of 2102 known protein interactions and 1975 protein pairs that do not interact in GPS-Prot. GPS-Prot requires a primary protein, which then returns an interaction network based on previously reported interactions. Our inclusion criteria required orthologs for each protein to be annotated in at least 100 species, and non-interacting pairs were checked for both primary and secondary interactions (i.e., inclusion in the non-interacting set required protein pairs to be at least three degrees removed from a known interaction). Table S3 provides a complete list of protein pairs that were included in our validation set, which is based on the interaction networks from the randomly chosen proteins associated with Alzheimer’s disease.

We used all available data in the STRING database [6,7,8,9,10,11,12,13,14,15,16] as a representative sample for bacteria protein interaction. The STRING database contains different groups of proteins called Clusters of Orthologous Genes (COGs). These COGs are derived and assigned an interaction score based on a combination of experimental data, database mining, and their own computational analysis. We downloaded the STRING dataset with accompanying COG scores representing the strength of the interactions between proteins in each COG in December 2020. We then filtered this dataset for COGs with the highest possible score and those with the lowest possible score. We converted those high- and low-interacting COG identifiers into their corresponding lists of protein names using the provided conversion table on the STRING website. We then filtered for proteins with orthologs spanning at least 100 species, which identified 233 protein pairs with high interaction scores and 252 protein pairs with low interaction scores. Those protein pairs were respectively labeled as groups that were “likely to interact” and “unlikely to interact.” All protein interactions used in these analyses are included in our GitHub repository at https://github.com/kauwelab/pic/tree/master/supporting_work/exampleData.

Hyperparameters for the minimum $p_{\mathrm{XY}}\left(x,y\right)$ and minimum $p_{X}\left(x\right)$ values were tuned for vertebrates and bacteria separately using their respective datasets by calculating a two-sample *t*-test, Cohen’s D, and area under the curve statistic for a ROC curve using various combinations of values. The $p_{\mathrm{XY}}\left(x,y\right)$ parameter was tuned from 0.1–0.5, and $p_{X}\left(x\right)\;$ranged from 0.0–0.35, which fully encapsulated the maximum values on the heatmaps for each test statistic. Since each set of hyperparameters was run on each protein pair, 1476 combinations of hyperparameter filters were run across all bacteria and vertebrate protein interactions, which required substantial computational resources. Therefore, we did not explore the parameter search space beyond the clear maximum values that were evident in the heatmaps. Precision was prioritized over a recall when establishing default parameters, and the PIC uses default parameters that produce the highest precision while maintaining at least 20% recall. The scripts used for these analyses are available at https://github.com/kauwelab/pic/tree/master/supporting_work/verification_I.

### 2.4. Probability Thresholds

The PIC also aims to provide the likelihood that a predicted interaction is a true interaction. After fine-tuning the hyperparameters, we established 20 bins of scores for both the known and unknown interactions ranging from zero to the maximum reported score for each of the bacteria and vertebrate sets. We then calculated the probability that each bin identified known interacting pairs. Bins where the probability of identifying known interactions was ≤50% (i.e., random) were merged since our validation method is more likely to identify false negatives (e.g., protein interactions that have not yet been identified) than false positives. These probabilities are reported on the website as well as the command-line interface to help users contextualize their scores against our training data.

### 2.5. Verification II

We conducted pairwise proteome-wide mutual information calculations to identify proteins that were most likely to interact with 62 proteins previously prioritized in Alzheimer’s disease genome-wide association studies (GWAS) [5,48,49]. Table S4 shows those previously prioritized proteins, the number of species with that annotated protein sequence, and the number of proteins containing sufficient ortholog annotations to run the protein interactions calculator (i.e., at least 100 species contained ortholog annotations in RefSeq [50,51] for both the query and subject sequences). The longest isoform of each protein was always chosen to encapsulate the most possible interactions. Those filters narrowed our analyses to include 37 of the 62 proteins (containing 40 risk loci), which were compared to each other as well as 8610 other proteins. In total, 57,561 total pairwise comparisons were conducted. Of those pairwise comparisons, 135 were previously reported to interact in GPS-Prot or the STRING database.

We calculated the probability that each calculated pairwise protein interaction score was a true interaction based on the probabilities from our vertebrate test set. We also used fine-tuned filters from our previous verification tests to evaluate the Alzheimer’s disease proteins. Finally, we report the identified proteins that were previously implicated in Alzheimer’s disease, the proteins that were previously reported in GPS-Prot [17], and the residues that are most likely to be functionally conserved between the interacting proteins.

### 2.6. Comparisons to Other Calculators

#### 2.6.1. Overview of Other Available Calculators

BIS2Analyzer [31] is a powerful tool that finds clusters of coevolving residues within proteins. It has aided in discovering features of the fusion mechanism of the Hepatitis C virus [52] and analyzing the connection between protein disorder and the precision of the retinoic acid receptor’s (RAR’s) repressive activity [53]. BIS2Analyzer provides users with several options for how the input data will be prepared and processed and which of the available algorithms will be used. This functionality results in a powerful tool with a high start-up cost because it requires knowledge and understanding of the input files as well as all available BIS2Analyzer functionality. While useful in a range of contexts, BIS2Analyzer is not well-suited nor designed for identifying potential relationships or interacting clusters between proteins. Even though the website notes that it is possible to compare two proteins to one another, the process to do so involves concatenating protein sequences onto one another to create a mega sequence that is analyzed as if it were one protein. Then, once the output is received, the user is responsible for sorting between inter- and intra-protein interactions. Even after sorting those interactions, it is still difficult to understand whether the two proteins interact because the results are not normalized against a standard population. As BIS2Analyzer was designed for single protein analyses, and it is assumed that a single protein is related to itself, there is no clear indication as to whether a relationship is likely or unlikely to be present. Although BIS2Analyzer has a comprehensive web interface, a stand-alone command line interface to perform bulk analyses is not available on GitHub. Furthermore, BIS2Analyzer is not a fast tool appropriate for bulk data analyses to explore potential co-evolution [31].

MISTIC2 [34] identifies co-evolving residues within a protein and is often used to model the protein folding structure [54] but has also been used to identify likely functional mutations [55]. It accepts a single multiple-sequence alignment as input, uses corrected mutual information to determine the likely folding structure, and identifies interaction networks within the protein. This tool is also intended for only a single protein. While concatenating two proteins may also be used with MISTIC2, disentangling inter- from intra-protein results is challenging and leads to potentially erroneous interpretation of the results. As with BIS2Analyzer, no command line interface is available for MISTIC2, and bulk analyses are not possible [34].

Some more recent protein functional calculators, such as AlphaFold [44,56,57], focus on protein folding, which can then be used to predict functional interactions. While protein structure is crucial to the ability of proteins to interact physically, it does not consider evolutionary conservation and does not provide residue-specific resolution of interaction predictions. Further, AlphaFold has no information on intra-protein interactions, only single protein folding structures. Other mutual information-based tools are now deprecated and lead to an error page [32] or never return results from submitted jobs [33].

#### 2.6.2. Runtime Comparisons

Since available mutual information calculators are not able to quickly process bulk protein interactions, we compared the runtimes of the PIC, MISTIC2, and BIS2Analyzer using five randomly chosen proteins listed in Table S5. MISTIC2 and BIS2Analyzer were both developed for intra-protein analyses, so these runtime comparisons were completed with a single protein. A single protein may be run on the PIC by simply inputting the same file for protein 1 and protein 2. Since the jobs for MISTIC2 and BIS2Analyzer needed to be submitted to a server, wait in a job queue, have the results returned by email, and would take several minutes, we based these times on the time submitted or indicated start time in an email and the time at which an email was received to notify us of job completion. Because the PIC finishes most jobs in under a minute, its times were measured from the time of submission to the time of completion.

#### 2.6.3. Precision and Recall Comparisons

Because the PIC is used to identify potential new interactions and not simply predict known interactions, we compared how often our calculator identified the same interactions as BIS2Analyzer as well as how often those predictions are correct. We did not compare the PIC to MISTIC2 because the MISTIC2 compute time makes it computationally intractable to perform these types of bulk analyses.

BIS2Analyzer was meant to be run on single protein sequences, so rather than reporting the likelihood of protein interactions, it instead reports which amino acid residues interact with one another in different residue clusters. To calculate mutual information between two proteins using BIS2Analyzer, the BIS2Analyzer website suggests concatenating protein sequences together. Then, if clusters are found to have positions from both proteins, those two proteins are classified as interacting. In contrast, the PIC results were assumed to interact if the chance of interaction was above 50%.

Batch computing is not possible on BIS2Analyzer, so we ran the 135 known interactions and a randomly selected sample of 100 of the 57,426 unknown protein interactions to compare BIS2Analyzer to the PIC. We do not report 20 known interactions and nine unknown interactions in our final BIS2Analyzer results because they did not produce any output when run on the BIS2Analyzer.

## 3. Results

### 3.1. Verification I

**Figure 1:**
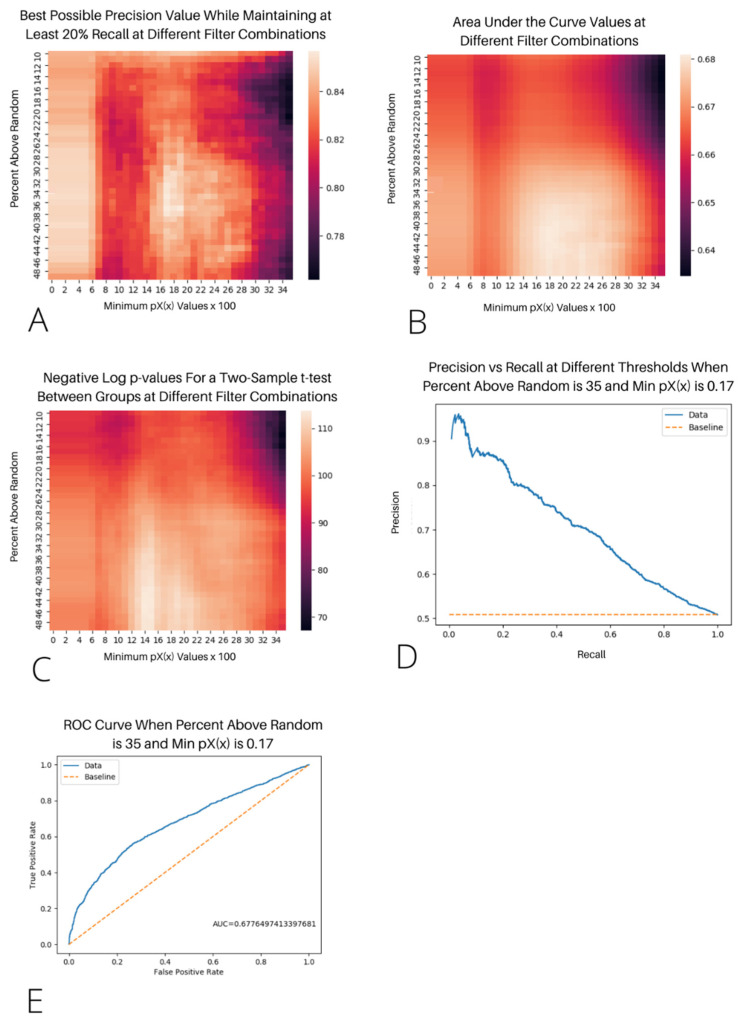
The results of different statistical tests when comparing protein pairs that are known to interact versus those that have not been proven to interact to show trends in the data for the vertebrate dataset. (**A**) A heatmap of the best possible precision scores obtained by comparing the known-to-interact and unproven-to-interact scores for each filter combination while still maintaining a recall of at least 20%. (**B**) A heatmap of the values of the area under the curve from comparing the known-to-interact and unproven-to-interact scores for each filter combination. (**C**) A heatmap of the negative log of the *p*-values from a two-sample *t*-test by comparing the known-to-interact and unproven-to-interact scores for each filter combination. Note that while each combination returned a significant *p*-value, some combinations were more significant than others. (**D**) A graph of all precision and recall values for the results from a min $p_{X}\left(x\right)$ of 0.17 and a minimum percent above random for $p_{\mathrm{XY}}\left(x,y\right)$ of 35%, which were determined to be the optimal filters due to the resulting high precisions. (**E**) The receiver operating characteristic (ROC) curve from comparing the known-to-interact and unproven-to-interact scores for each filter combination.

We identified the optimal filters for min $p_{X}\left(x\right)$ and the percentage above the random by testing all of the combinations of min $p_{X}\left(x\right)$ from 0–0.35, incrementing by 0.01, and the percentage above the random from 10–49%, incrementing by 1.0% for a total of 1440 setting combinations. This process was repeated for both bacteria and humans. We assessed the model accuracy by maximizing the area under the curve (AUC) and precision while maintaining at least a 20% recall (see Figure 1A,B). The AUC measures how well the algorithm performs over the range of the dataset, while the precision measures how well the algorithm can perform in the most optimal scenario while correctly labeling at least 20% of the possible interactions. We found that both the precision and AUC were maximized when the min $p_{X}\left(x\right)$ = 0.17 and the percent above the random = 35 for the vertebrates (see Figure 1D,E) and when the min $p_{X}\left(x\right)$ = 0.29 and the percent above the random = 22 for the bacteria (see Figure S3). The AUC for humans and bacteria were 0.6617 and 0.7044, respectively. The highest precision for predicting at least 20% of the possible interactions was 0.8478 and 0.7391 for humans and bacteria, respectively. The precision-recall curve for the most optimal filters in humans shows a steep decline in the precision as the recall increases, with the precision being 0.7 when the recall is 0.4 and 0.6 when the recall is 0.75 (see Figure 1D).

We also performed a two-sample t-test on the results using each filter combination to assess the differences between the interacting and unproven-to-interact groups. At each filter combination, the results were significant at the α = 0.05 level and were even significant using the Bonferroni correction for multiple testing (α = 0.05/2880 = 1.74 × 10^−5^). For the bacteria, *p* ranged from 5.77 × 10^−38^ to 5.15 × 10^−7^, and for the vertebrates, *p* ranged from 1.68 × 10^−114^ to 7.32 × 10^−68^. At the chosen filter settings, the two-sample *t*-tests yielded *p =* 1.03 × 10^−108^ for the vertebrates and *p* = 3.29 × 10^−20^ for the bacteria.

### 3.2. Probability Thresholds

Since each potential pairwise protein interaction is given a standardized score that facilitates direct comparisons between other protein interactions, we were able to establish probabilities of a true interaction based on certain thresholds. Table 1 and Table 2, respectively, show the score thresholds for vertebrates and bacteria.

### 3.3. Verification II

Using the vertebrate thresholds, we evaluated all of the potential interactions between Alzheimer’s disease-associated proteins and 8608 proteins that had ortholog annotations spanning at least 100 species. More information about these pairings can be found in Table S4. We found 1933 interactions that exceeded a 95% likelihood of interaction, with 98.65% of those interactions being previously undocumented in GPS-Prot. Table S5 shows the number of interactions exceeding each threshold and the percentage of those interactions that were previously reported in GPS-Prot or STRING.

Of the 135 known interactions in the Alzheimer’s disease dataset, BIS2Analyzer identified 88 of them as interacting and failed to run to completion on 20 pairs. The PIC identified 64 as interacting. Of the 91 pairs not known to interact, BIS2Analyzer identified 70 as interacting, whereas the PIC identified only 24 (*p* = 1.27 × 10^−11^). 

We allow users to query all Alzheimer’s disease interactions at https://pic.byu.edu/. The interactive user interface auto-completes gene names based on the interactions present in the dataset. The website then displays the highest interacting residue positions in each protein sequence, the probability that the two proteins interact, and the normalized interaction score. Additionally, an interactive online version of the algorithm allows users to run the PIC on their own datasets. Users can input two multiple sequence protein alignments containing at least 100 overlapping header names (i.e., species), and the online algorithm will calculate the probability that the two proteins interact. The website will then automatically produce a heatmap of all of the residue interactions and display the highest residue–residue interaction (see Figure X for an example of the website output). A command-line version of the algorithm is also available at https://github.com/kauwelab/pic to facilitate high-throughput programmatic analyses.

### 3.4. Runtime Comparisons to Other Calculators

The PIC is significantly more computationally efficient for calculating pairwise protein–protein interactions than other available software packages. For instance, MISTIC2 and BIS2Analyzer calculate mutual information from a single protein sequence but do not allow users to calculate mutual information between protein sequences. or doing so requires several extra data preparation and interpretation steps. Past analyses combined the protein sequences by adding a string of 20 amino acids at the end of the first alignment and then concatenating the second multiple sequence alignment to that of the first alignment. While that method allows mutual information to be calculated between proteins, it requires extra programmatic steps, and the algorithm is very inefficient comparing amino acids that do not need to be compared.

Further, when compared to other developed tools, even when ignoring the barriers to comparing two different proteins, the PIC runs significantly faster. As MISTIC2 is unable to perform bulk analysis, we report the runtimes of five randomly selected intra-protein interactions to assess the differences in the runtime (See Table 3).

## 4. Discussion

The PIC is a fast mutual information calculator used to identify the residue-specific coevolution between two proteins. We envision that the PIC will help researchers prioritize potential protein interactions for additional biological validation. While we recognize that low mutual information alone is not enough to exclude a potential interaction, a high mutual information score indicates that residues have coevolved through functional interactions.

The PIC improves on previous calculators by providing both online and command-line interfaces with standardized probabilities for users to interpret scores. Additionally, the PIC was developed for large-scale analyses, which enables proteome-wide comparisons that are not currently possible using available calculators, such as BIS2Analyzer and MISTIC. While BIS2Analyzer identified more known interactions than the PIC, it had a much higher false positive rate, which can be misleading. The PIC, on the other hand, was fine-tuned for increased precision, which gives users higher confidence in the reported values. The optional parameters and comprehensive comparisons of different hyperparameters allow users to adapt the PIC to their own research in ways that are not possible with the other available calculators.

Additionally, we present the PIC as an open-source software package to facilitate the use of mutual information in future protein interaction calculators and models. Since the PIC identifies residue-specific interactions, it has the potential to improve the resolution of machine learning algorithms that often analyze broader features. Since the PIC was specifically developed to analyze inter-protein interactions, it is much more user-friendly for these types of analyses than available software that analyze intra-protein interactions.

We validated the PIC with proteins that were previously implicated in Alzheimer’s disease because the etiology of Alzheimer’s disease remains unclear, and the heterogeneity contributes to the current dearth of viable treatments. We hypothesized that secondary interactions between the primary disease-associated loci and coevolving residues on other proteins might mask the effects of interacting proteins on Alzheimer’s disease. Therefore, our proteome-wide analysis of coevolving residues prioritizes potential protein interactions for follow-up studies. Since the PIC ranks potential protein interactions by their probability of being true interactions, we propose that the identified interactions with the highest scores should be prioritized for additional validation.

## 5. Conclusions

The PIC is currently the best mutual information calculator to identify the residue-specific coevolution between two different proteins. The PIC was designed to reduce the computational complexity of these types of analyses and provide a user-friendly online interface to facilitate high-throughput protein interaction calculations across various domains. We specifically report proteins that the PIC prioritized as most likely to interact with Alzheimer’s disease-associated proteins. Those predictions provide users with a residue-specific resolution that indicates specific amino acids that are most likely to interact with each other. Additional scoring indicates the probability that the reported interactions are true interactions based on our validation set. The biological validation of the highest scoring interactions is warranted and may lead to a better understanding of the etiology of Alzheimer’s disease. We anticipate that the PIC will be widely used as an additional prioritization filter to increase the protein interaction resolution and identify functional interactions in disease-associated proteins, which may lead to additional therapeutic targets.

## Figures and Tables

**Table 1:** The different probability thresholds for mutual information scores for vertebrates.

Vertebrate Mutual Information Score Thresholds												
Score	480.76	151.52	79.29	45.61	30.18	20.99	15.04	10.93	7.85	6.17	4.62	3.59
% chance of interaction	99.68	98.43	97.32	95.85	92.23	89.40	85.31	80.93	75.51	69.99	63.81	55.96

**Table 2:** The different probability thresholds for mutual information scores for bacteria.

Bacteria Mutual Information Score Thresholds													
Score	44.40	32.01	23.93	20.14	18.26	17.03	15.00	13.19	11.07	10.43	9.46	8.34	7.09
% chance of interaction	98.75	96.56	75.58	95.09	90.45	89.89	84.70	79.83	75.61	72.36	66.89	61.25	56.09

**Table 3:** Runtimes in minutes for three different calculators, including our own, on 5 different randomly selected proteins. Though our calculator is designed for intra-protein interactions, it can easily be used for inter-protein interactions. The reverse is not true for the other calculators. Hence, this test was conducted on inter-protein relationships. Note that runtimes are approximate and based on email notifications of when jobs started and finished for BIS2Analyzer and MISTIC2.

Protein	BIS2 Analyzer Runtime (Minutes)	MISTIC2 Runtime (Minutes)	PIC Runtime (Minutes)
LMNA	3	20	0.43
CDK2	0.5	3	0.12
RB1	4	36	0.62
AR	2.5	30	0.65
ELL	13	6	0.13

## Data Availability

All scripts used to replicate this work are available at https://github.com/kauwelab/pic. An online version of the algorithm is publicly available at https://pic.byu.edu.

## Supplementary Materials

The following supporting information can be downloaded at: https://www.mdpi.com/article/10.3390/genes13081346/s1, Figure S1: Partial Mutual Information Calculation as it Applies to an MSA; Figure S2: Measures of Validity at Each Filter Combination For Vertebrates; Figure S3: Measures of Validity at Each Filter Combination For Bacteria; Text S1: Explanation of Partial Mutual Information Filters; Equation S1: Partial Mutual Information; Equation S2: Evaluation of Partial Mutual Information for Perfectly Random Co-occurrence; Equation S3: Evaluation of Partial Mutual Information with low individual frequencies; Table S1: Description of Primary Vertebrate Proteins for Interacting Pairs; Table S2: Description of All Interacting Vertebrate Proteins; Table S3: Description of Primary Vertebrate Proteins Used in Non-Interacting Pairs; Table S4: Alzheimer’s disease Analysis Information by Protein; Table S5: Potential protein interactions with Alzheimer’s disease-associated proteins.
