# Supplementary material for: Web-Based Protein Interactions Calculator Identifies Likely Proteome Coevolution with Alzheimer’s Disease-Associated Proteins
Source: Genes (Basel). 2022 Jul 27;13(8):1346. doi: 10.3390/genes13081346 (PMC9407263; doi:10.3390/genes13081346)
Supplement: Supplementary file 1 [file genes-13-01346-s001.zip › genes-1833516-supplementary.pdf]

Supplemental Material: Evaluating Protein Coevolution with a Fast Web-based Protein Interactions Calculator

Katrisa M. Ward[1], Brandon D. Pickett[1], Mark T. W. Ebbert[2,3,4], John S. K. Kauwe[1,+], Justin B. Miller[2,3,5,†,*]

[1]Department of Biology, Brigham Young University, Provo, UT 84602, USA; katrisa14@gmail.com (K.M.W.); pickettbd@byu.edu (B.D.P.); kauwe@byu.edu (J.S.K.K.)
[2]Sanders-Brown Center on Aging, University of Kentucky, Lexington, KY 40536, USA; mark.ebbert@uky.edu
[3]Division of Biomedical Informatics, Department of Internal Medicine, University of Kentucky, Lexington, KY 40506, USA
[4]Department of Neuroscience, University of Kentucky, Lexington, KY 40506, USA
[5]Department of Pathology and Laboratory Medicine, University of Kentucky, Lexington, KY 40506, USA

[*]Corresponding author: justin.miller@uky.edu
[†]These authors contributed equally to this work.

Table of Contents

Supplementary Figures 3
Figure S1 – Partial Mutual Information Calculation as it Applies to an MSA 3
Figure S2 – Measures of Validity at Each Filter Combination For Vertebrates 4
Figure S3 – Measures of Validity at Each Filter Combination For Bacteria 5
Supplementary Text 6
Explanation of Partial Mutual Information Filters 6
Supplementary Equations 7
Equation S1 –Partial Mutual Information 7
Equation S2 – Evaluation of Partial Mutual Information for Perfectly Random Co-occurrence 7
Equation S3 – Evaluation of Partial Mutual Information with low individual frequencies 7
Supplementary Tables 8
Table S1 – Description of Primary Vertebrate Proteins for Interacting Pairs 8
Table S2 – Description of All Interacting Vertebrate Proteins 8
Table S3 – Description of Primary Vertebrate Proteins Used in Non-Interacting Pairs 33
Table S4 – Alzheimer's disease Analysis Information by Protein 33
Table S5 – Potential protein interactions with Alzheimer’s disease-associated proteins 35

Supplementary Figures

Figure S1 – Partial Mutual Information Calculation as it Applies to an MSA

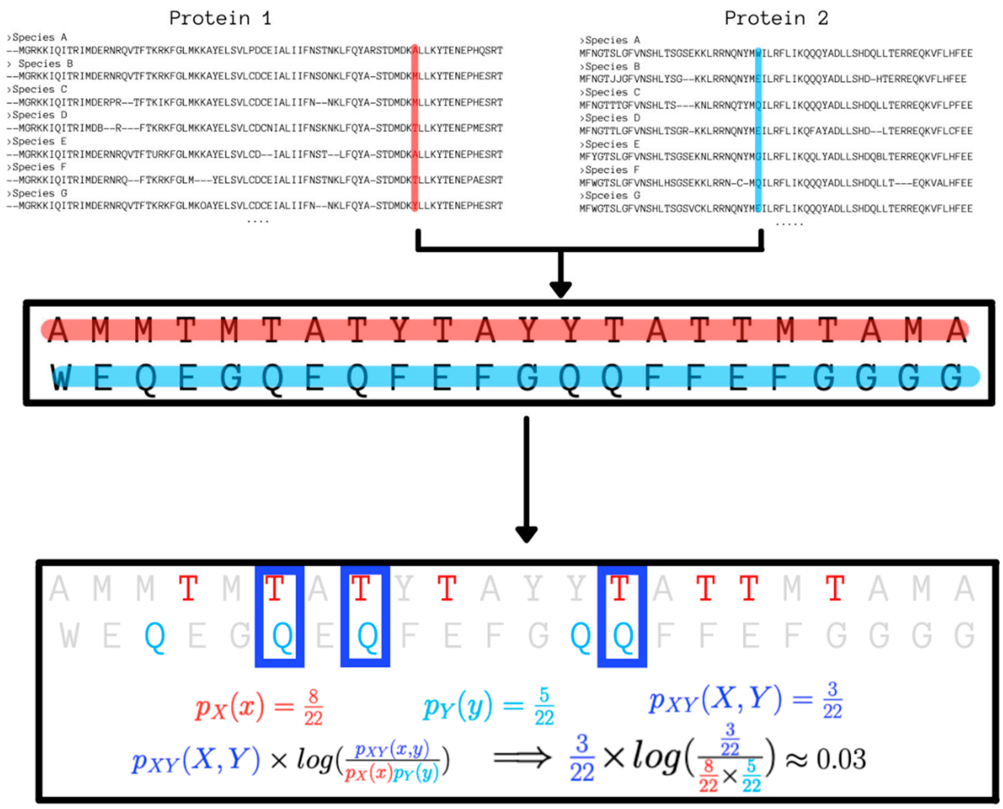

Figure S1 - Mutual information is used to detect dependence between any two discrete variables, here called X and Y, by comparing the individual frequencies of values with the frequency of their joint occurrences. For each combination of values from X and Y, a partial mutual information (pMI) score is calculated, and then each pMI score is summed to get the overall mutual information score for the variable pair. In eq. S1 and in the above figure, pX(x) is the frequency with which variable X takes on the value x, pY(y) is the frequency with which the variable Y takes on the value y and pXY(x,y) is the frequency of the co-occurrence of the two values.

Mutual information may be applied to multi-sequence alignments of proteins. Here, a single position on a protein represents a discrete variable. Depending on the species, one of several different amino acids may occur at that position. The amino acids themselves are then the values, and the co-occurrence of values is represented by two values at different positions in the same species. Mutual information may be calculated between any two protein positions, including from within the same protein, but for our purposes we calculate the mutual information between each pair of positions between two different proteins. Hence, several mutual information scores are calculated for each protein pair.

The process of calculating a single pMI score in this context is shown. The two positions spanning two different proteins, highlighted above in red and blue, are the two variables, X and Y. We show an example of calculating the pMI score for T from position 1 and Q from position 2. T occurs 8 times in 22

species on position 1 while Q occurs 5 times in 22 species. T_1 and Q_2 occur in the same species 3 times. Therefore, the pMI score for T_1 and Q_2 is about 0.03.

Figure S2 – Measures of Validity at Each Filter Combination For Vertebrates

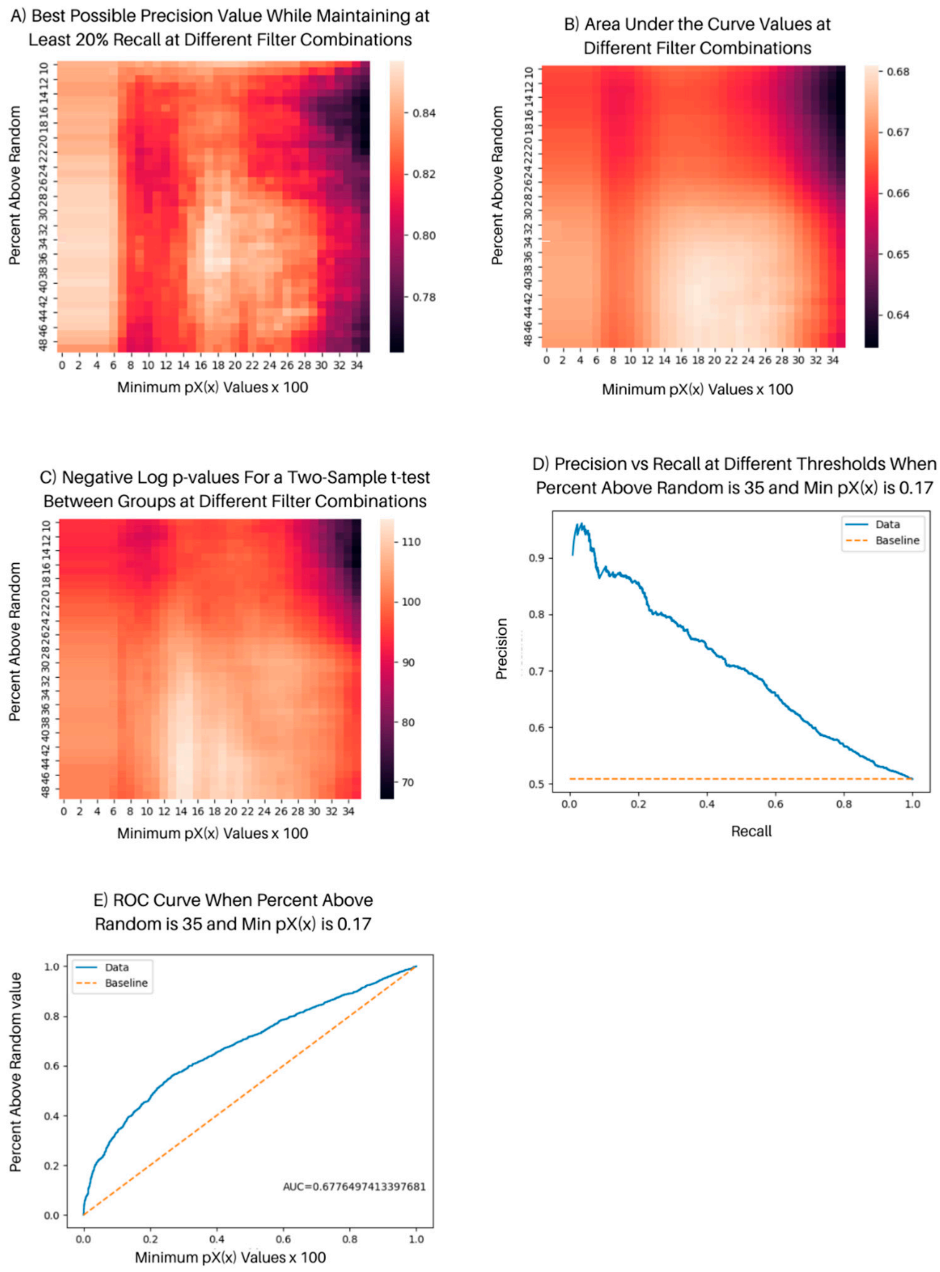

Figure S3 – Measures of Validity at Each Filter Combination For Bacteria

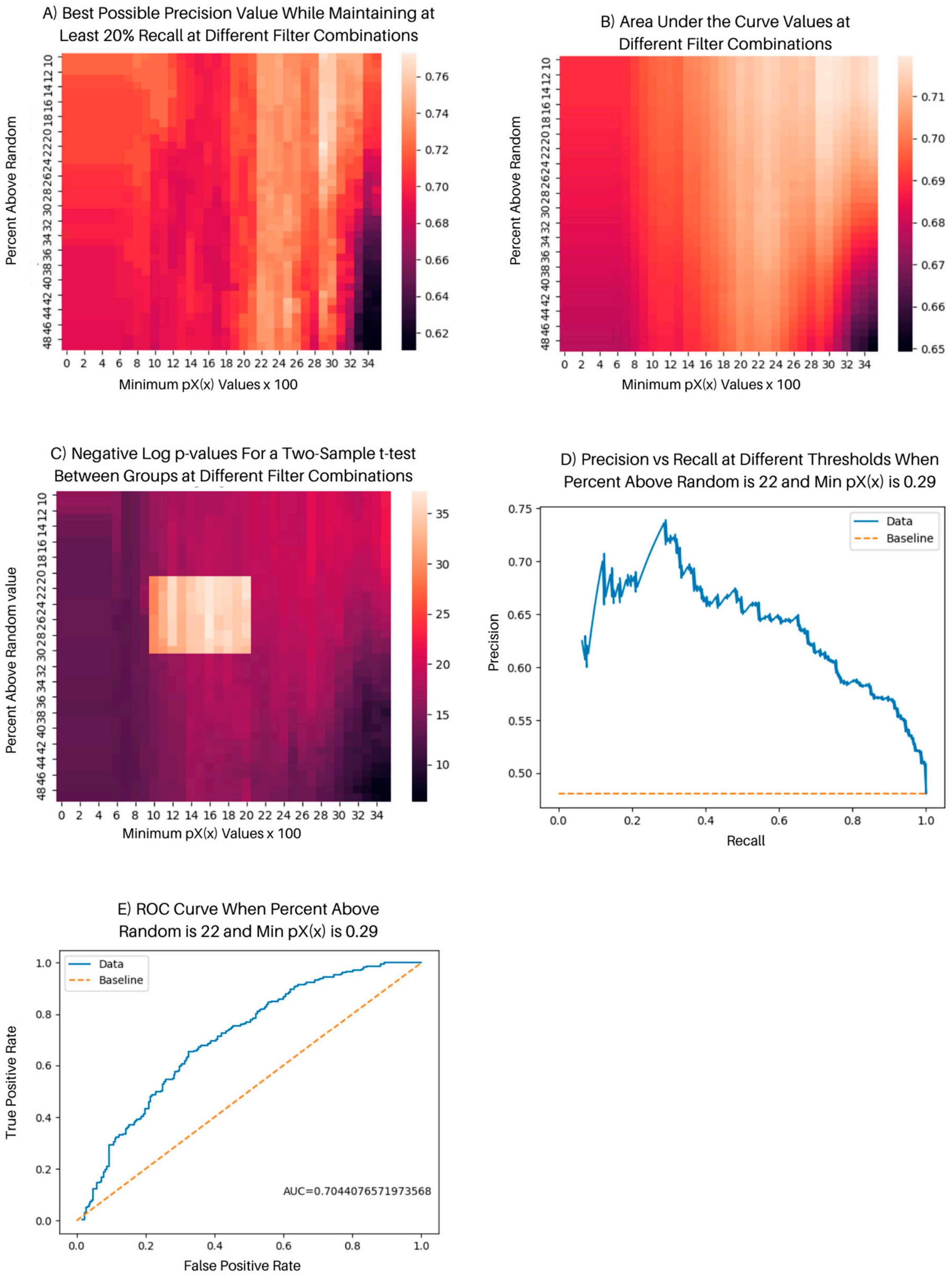

Supplementary Text

Explanation of Partial Mutual Information Filters

Mutual information is a measure of the dependence of two variables. It is calculated by comparing the frequency of values from each individual variable to how often variable pairs co-occur. Therefore, mutual information measures how well you can predict the value of one variable by knowing the value of the other variable. Figure S1 explains how a mutual information score is a summation of several partial mutual information scores, summed across all possible values.

Note that the two lists in Figure S1 were randomly generated. Therefore, mutual information should not indicate a relationship between the two lists. Mutual information accounts for randomness by calculating the probability of two independent events co-occurring (i.e., the probability of one event occurring multiplied by the probability of the other event occurring). Therefore, if X and Y are unrelated, independent lists, then pXY(x,y) should be equal to pX(x) * pY(y). Equation S2 shows that if the two values are equivalent, then the logarithm evaluates to zero, which cancels the partial mutual information score.

However, in the randomly generated example, T and Q do not co-occur at a perfectly random rate, and the partial mutual information score is positive and non-zero. Although the score, 0.03, is relatively low, it is not insignificant to calculated protein coevolution scores. Therefore, we altered the mutual information algorithm by summing only partial mutual information scores where the value of p(X,Y)(xy) is a certain percentage or more above random — rather than just strictly above random. We found that results were most accurate if this threshold was set at 35% above random, although users can define this parameter.

Adjusting the threshold for randomness alone was not sufficient to reduce the background noise often discussed in conjunction with mutual information and calculate the most accurate results. Consider an example in which two values co-occur, yet occur only once in their respective lists of length 40. These conditions will yield a relatively high partial mutual information score (see Equation S3).
At first consideration, the high score might seem warranted, as the probability of co-occurrence is exceptionally low. However, that probability is artificially deflated because protein coevolution calculations are based on incomplete datasets. Multiple sequence alignments do not contain every ortholog and isoform of a particular protein. Therefore, this hypothetical co-occurrence is likely caused by a phylogenetic sampling bias where species selection inflates the mutual information score. To mitigate this phylogenetic bias, we required that pX(x) and pY(y) values also be above a certain threshold. We found that results were most accurate when this threshold was set to 0.17

As a third and final measure, each mutual information score was divided by the average mutual information score for the comparison, similar to what is done with the Average Product Correction used by other calculators. Finally, scores were multiplied by 10,000 for readability since the values were generally low.

Supplementary Equations

Equation S1 –Partial Mutual Information

$$MI(X,Y) = \sum_{x \epsilon X} \sum_{y \epsilon Y} p_{XY}(x,y) \times \log\left(\frac{p_{XY}(x,y)}{p_X(x) p_Y(y)}\right)$$

Equation S2 – Evaluation of Partial Mutual Information for Perfectly Random Co-occurrence

$$p_{XY}(x,y) = p_X(x) \times p_Y(y) \Rightarrow \mathrm{pMI} = \log\left(\frac{p_{XY}(x,y)}{p_X(x) \times p_Y(y)}\right) = \log(1) = 0$$

Equation S3 – Evaluation of Partial Mutual Information with low individual frequencies

$$p_{XY}(x,y) = p_X(x) = p_Y(y) = \frac{1}{40} \Rightarrow pMI = \frac{1}{40} \times \log\left(\frac{\frac{1}{40}}{\frac{1}{40} \times \frac{1}{40}}\right) \approx 0.04$$

Supplementary Tables

Table S1 – Description of Primary Vertebrate Proteins for Interacting Pairs

Protein	Number of direct pairings
**DMD**	49
**BRCA1**	164
**ASPM**	71
**APP**	109
**PSEN1**	100
**RBM45**	37
**LRRK2**	49

Table S2 – Description of All Interacting Vertebrate Proteins

Protein	Primary Protein(s)	Degrees of separation from primary protein	# of pairings
**MDC1**	BRCA1	1	21
**SQSTM1**	RBM45	1	8
**ACTA1**	DMD	1	8
**RPS14**	APP	1	10
**SMAD2**	BRCA1	1	7
**CSTF1**	BRCA1	1	4
**SLC25A4**	LRRK2	1	1
**PRKCSH**	DMD/APP	2	1
**FNDC3A**	RBM45	1	1
**UMPS**	PSEN1	1	2
**HSP90AA1**	APP/LRRK2	1	8
**UTRN**	DMD	1	17
**DOT1L**	RBM45	1	1
**RPP38**	ASPM	1	2
**CDC5L**	BRCA1/RBM45	1	12
**CDK8**	BRCA1/RBM45	1	4

**LARP1B**	APP/DMD	1	2
**SERPINH1**	BRCA1	1	5
**SMC1A**	BRCA1	1	3
**MANSC1**	APP	2	1
**MYC**	ASPM/BRCA1	1	34
**TP73**	ASPM/BRCA1/LRRK2	2	4
**KEAP1**	BRCA1/RBM45	1	10
**PMS1**	BRCA1	2	2
**ART3**	APP/PSEN1	2	1
**CEP55**	APP/PSEN1	2	3
**TGFB2**	APP	1	2
**VCP**	ASPM/BRCA1	1	23
**GRB2**	ASPM/BRCA1/LRRK2	2	12
**BABAM1**	BRCA1	1	3
**MTCH1**	PSEN1	1	2
**LCAT**	DMD/APP/PSEN1	5	1
**BACE1**	APP/PSEN1	1	3
**BRAT1**	BRCA1	1	1
**E2F1**	BRCA1	1	7
**MYO1C**	LRRK2	1	1
**HAUS1**	DMD	1	3
**FANCA**	BRCA1	1	4
**SFTPC**	APP	1	1
**VDAC3**	BRCA1/APP/LRRK2	3	2
**HSPB8**	BRCA1/DMD	2	1
**MYD88**	APP/PSEN1	2	5

**SF3B2**	APP	1	5
**BAP1**	BRCA1	1	1
**SNTG2**	DMD	1	8
**ADAMTSL5**	APP/PSEN1	3	2
**NUFIP1**	BRCA1	1	1
**GAK**	LRRK2	1	2
**HMG20B**	BRCA1/DMD	2	2
**PLXDC2**	PSEN1	1	2
**CD81**	DMD/APP/PSEN1	2	6
**SPI1**	APP/PSEN1	3	2
**AAMP**	APP/PSEN1	2	2
**VPS35**	APP/PSEN1	2	2
**RAD50**	BRCA1	1	6
**LRP1**	APP	1	5
**RPA2**	BRCA1	1	15
**HSPG2**	APP/PSEN1	3	1
**NDUFA9**	PSEN1	1	3
**PPP5C**	APP	2	1
**PDCD4**	PSEN1	1	8
**APOA1**	APP	1	2
**ATF1**	BRCA1	1	3
**DGKZ**	DMD	1	7
**TRIM25**	APP/DMD	1	13
**VASN**	PSEN1	1	3
**SNTG1**	DMD	1	4
**NUP133**	LRRK2	1	1

**HOXB13**	APP/PSEN1	3	1
**MGMT**	BRCA1/LRRK2	2	4
**APOA2**	DMD/APP/PSEN1	4	2
**ZRANB2**	LRRK2	1	1
**ARFGAP1**	APP/LRRK2	1	3
**ATXN7**	APP/PSEN1	3	3
**ANK2**	DMD	1	1
**PLK1**	ASPM/BRCA1	1	18
**TOP2A**	BRCA1	1	9
**PPP1CB**	BRCA1	1	2
**FEZ1**	ASPM	1	4
**SMC3**	BRCA1	1	14
**TARDBP**	BRCA1/RBM45	1	10
**LONP1**	LRRK2/APP/PSEN1	2	5
**ELL**	ASPM	1	3
**SMARCA2**	BRCA1	1	4
**CRYAB**	APP	1	2
**NOS3**	APP/PSEN1	1	5
**PMS2**	BRCA1	1	7
**PEX19**	ASPM	1	2
**NPM1**	BRCA1/ASPM	1	18
**TTR**	APP	1	2
**CTNND1**	APP/PSEN1	1	9
**ELAVL2**	RBM45	1	8
**JUN**	BRCA1	1	20
**PGR**	BRCA1	1	4

**STX1A**	BRCA1/APP	2	2
**PCOLCE**	APP	1	1
**POLR2A**	BRCA1	1	9
**POLH**	BRCA1	1	4
**CIB1**	PSEN1	1	2
**DEF6**	RBM45	1	1
**TUBGCP2**	ASPM/BRCA1	2	8
**CCND1**	BRCA1	1	8
**RAB5B**	LRRK2	1	1
**RPS26**	APP	1	8
**HSP90AB1**	LRRK2	1	8
**SSR3**	ASPM	1	1
**FGF13**	APP/PSEN1	1	2
**FANCE**	BRCA1	1	4
**RNF168**	BRCA1	1	3
**UBE2L3**	BRCA1	1	1
**NGFR**	APP	1	1
**KATNAL2**	ASPM	1	5
**TMED10**	PSEN1	1	5
**ELAVL1**	BRCA1/APP/PSEN1	2	20
**CSTF2**	BRCA1	1	3
**FOXJ1**	ASPM	1	2
**TPM1**	LRRK2/APP/PSEN1	2	7
**CDH2**	PSEN1	1	8
**UBE3A**	ASPM/BRCA1/APP/LRRK2	2	7
**BRCA1**	BRCA1	self	164

**DMD**	DMD	self	49
**TUBGCP3**	ASPM	1	7
**PLEKHA6**	APP/PSEN1	2	1
**KIAA2013**	RBM45	1	2
**UBE2D2**	BRCA1	1	1
**PRDM1**	APP/PSEN1	3	1
**CEP78**	ASPM	1	1
**DTNB**	DMD	1	8
**KATNA1**	ASPM/RBM45/LRRK2	2	3
**CDH1**	PSEN1	1	9
**FOXRED2**	RBM45	1	1
**HNRNPC**	BRCA1	1	9
**NBN**	BRCA1	1	8
**ACACA**	BRCA1	1	1
**KIF4A**	BRCA1	1	2
**SHC1**	APP	1	2
**OS9**	ASPM	1	6
**NEDD4**	DMD	1	10
**TRAF6**	APP/PSEN1	1	27
**RPS8**	LRRK2	1	1
**YWHAG**	LRRK2	1	8
**ERN1**	PSEN1	1	2
**EIF3B**	BRCA1	1	1
**HAUS7**	ASPM	1	3
**KIF22**	BRCA1/RBM45	1	5
**APBB3**	APP	1	1

**LZTS2**	ASPM	1	4
**GFAP**	APP/PSEN1	1	7
**SKAP1**	ASPM	2	1
**SORL1**	APP	1	1
**BCL6**	APP/PSEN1	3	2
**THAP7**	ASPM	1	3
**ESR2**	BRCA1/RBM45	1	18
**DSG2**	PSEN1	1	3
**MAST1**	APP	1	10
**DCN**	APP/PSEN1	3	3
**RFXANK**	BRCA1	2	1
**NDUFS8**	PSEN1	1	3
**UBE2E1**	BRCA1	1	1
**TRAF3IP1**	APP/DMD	1	5
**TSPAN5**	APP	1	2
**HMGB1**	PSEN1	1	4
**MORF4L1**	BRCA1	1	6
**ZFC3H1**	APP/RBM45	1	3
**RHOB**	APP/PSEN1	3	1
**UBE2D3**	BRCA1	1	1
**KCNJ4**	DMD	1	2
**KCNIP3**	PSEN1	1	3
**NFX1**	DMD	1	3
**FLNB**	PSEN1	1	12
**TOP1**	APP/PSEN1	3	1
**RAB3A**	APP/PSEN1	1	6

**SYCP3**	BRCA1	2	1
**TCF4**	PSEN1	1	4
**NCL**	BRCA1/LRRK2	1	13
**RPL8**	PSEN1/BRCA1/DMD/APP/LRRK2	2	9
**RFC1**	BRCA1	1	4
**NEURL4**	ASPM/BRCA1/LRRK2	2	5
**NAE1**	APP	1	1
**TUBGCP4**	ASPM	1	6
**ANO3**	ASPM	1	1
**MAP3K5**	APP/DMD	1	5
**MSH2**	BRCA1	1	8
**EGFR**	APP	1	9
**JUNB**	BRCA1	1	3
**CAND1**	BRCA1/RBM45	1	12
**CSNK2B**	BRCA1	1	5
**KANK2**	PSEN1	1	2
**CTNNAL1**	DMD	1	6
**NFE2L2**	BRCA1/RBM45	1	6
**TRAF1**	RBM45	1	3
**DKK3**	ASPM	1	2
**RAD51C**	BRCA1	2	3
**DKK1**	RBM45	1	1
**PRKDC**	LRRK2	1	3
**APBA3**	APP	1	2
**YTHDC2**	APP/PSEN1	3	2
**SGCZ**	DMD	1	1

**ASPM**	ASPM	self	71
**PCNA**	BRCA1	1	23
**ETV7**	APP/PSEN1	3	1
**KATNB1**	ASPM	1	4
**TP53**	LRRK2/BRCA1	1	76
**CDH5**	ASPM	1	1
**AHNAK**	DMD/APP	2	1
**CCNA1**	BRCA1	1	4
**KPNA2**	BRCA1	1	8
**SNW1**	RBM45	1	15
**KAT5**	APP	1	2
**XRCC3**	BRCA1/DMD	2	6
**WDR54**	DMD	1	2
**ERLIN2**	PSEN1	1	5
**IPO9**	ASPM	1	8
**OXCT1**	PSEN1	1	2
**CDC42**	LRRK2	1	2
**ALB**	APP	1	6
**CDC25A**	APP/DMD	1	4
**VDAC1**	APP/LRRK2	2	7
**GSAP**	APP/PSEN1	1	4
**ELN**	APP/PSEN1	3	6
**SIRT2**	DMD	1	2
**HOMER3**	APP	1	1
**AGO2**	LRRK2	1	2
**PSMA7**	BRCA1	1	1

**KAT2B**	BRCA1/LRRK2	2	5
**ECSIT**	APP/PSEN1	1	24
**TRAFD1**	ASPM	1	3
**FBN2**	APP/PSEN1	3	7
**FBXW7**	PSEN1	1	5
**BLMH**	APP	1	1
**IER2**	BRCA1	2	1
**GCDH**	PSEN1	1	8
**ST13**	PSEN1	1	7
**CDK4**	ASPM/BRCA1	1	10
**KMT2D**	RBM45	1	3
**MAPK9**	PSEN1	1	8
**MAP2K6**	LRRK2	1	2
**HAUS4**	BRCA1/DMD	2	2
**CHEK2**	BRCA1	1	13
**SNRNP200**	BRCA1	1	2
**PSENEN**	PSEN1	1	7
**TMEM30A**	APP	1	1
**CDK9**	BRCA1	1	8
**WDR62**	ASPM/BRCA1	2	3
**NSL1**	ASPM/BRCA1/DMD/LRRK2	3	2
**CADPS2**	DMD	1	1
**ABCA1**	DMD	1	7
**RPL4**	LRRK2/DMD/APP/PSEN1	2	11
**RPS6KA1**	ASPM	1	4
**COL25A1**	APP	1	1

**SNTB1**	DMD	1	8
**PSMD8**	ASPM	1	5
**RCN1**	ASPM	1	7
**CREBBP**	BRCA1	1	18
**NAP1L4**	ASPM	1	6
**CRYZ**	ASPM	1	1
**CADPS**	DMD	1	1
**TERF1**	DMD	1	5
**HSPA8**	APP/BRCA1/LRRK2	1	29
**OSBPL1A**	DMD	1	2
**BATF**	APP/PSEN1	3	1
**APPBP2**	APP	1	1
**CHEK1**	BRCA1	1	6
**GSK3B**	APP/PSEN1	1	17
**HOXC11**	APP/PSEN1	3	1
**TNS1**	DMD	1	6
**MYO1D**	LRRK2	1	1
**FKBP4**	APP/PSEN1	3	1
**RPS28**	DMD/APP/PSEN1	2	9
**ENSA**	PSEN1	1	1
**ARHGEF7**	LRRK2	1	3
**LIG4**	ASPM	1	1
**MNAT1**	BRCA1	1	5
**PPM1G**	RBM45	1	4
**PSMD3**	BRCA1	1	9
**EVI5L**	APP/PSEN1	2	2

**CARM1**	APP/PSEN1	3	1
**SMYD2**	ASPM	1	2
**POU2F1**	BRCA1	1	5
**RPS7**	DMD/APP/LRRK2	2	8
**NOTCH1**	APP/PSEN1	1	9
**IARS2**	ASPM	1	3
**RIPK2**	APP/PSEN1	2	6
**ACTC1**	DMD	1	3
**PKP4**	PSEN1	1	6
**NPR1**	PSEN1	1	2
**ORC2**	BRCA1	1	2
**MCM2**	APP/DMD	1	11
**AR**	BRCA1	1	21
**RYR2**	PSEN1	1	1
**YWHAE**	LRRK2	1	7
**PSMB1**	PSEN1	1	5
**DTNA**	DMD/PSEN1	1	12
**FAF2**	PSEN1/APP/LRRK2	2	8
**BCL2**	PSEN1	1	6
**SKP2**	BRCA1	1	8
**SETX**	BRCA1	1	2
**GAN**	DMD	1	1
**SLC25A5**	LRRK2	1	1
**EFHD1**	PSEN1	1	1
**CDK5**	APP/PSEN1	1	10
**SUMO2**	ASPM/BRCA1	1	31

**ENO1**	BRCA1	1	3
**BCL2L1**	PSEN1	1	10
**MLH1**	BRCA1	1	6
**IPO7**	ASPM	1	6
**PRKCZ**	PSEN1	1	11
**L3MBTL1**	BRCA1	2	6
**E2F4**	BRCA1	1	4
**SPC24**	APP/ASPM	1	5
**CLU**	APP	1	1
**HMMR**	BRCA1	1	2
**UNK**	PSEN1	1	8
**STAT6**	APP/PSEN1	3	1
**TDP2**	PSEN1	1	2
**RPL10**	PSEN1	1	4
**TPM3**	APP/PSEN1	2	4
**PRMT1**	BRCA1	1	4
**LRRK1**	LRRK2	1	3
**CTSB**	APP	1	1
**ATR**	BRCA1	1	16
**IDE**	APP	1	1
**USP21**	RBM45	1	5
**PTGER4**	PSEN1	1	3
**HTRA1**	PSEN1/APP/LRRK2	2	1
**APOE**	APP/PSEN1	2	2
**GSN**	APP	1	1
**UBE2K**	BRCA1	1	2

**ECT2**	RBM45	1	4
**TMEM173**	RBM45	1	1
**CCNB1**	BRCA1	1	2
**DCTN3**	ASPM	1	1
**SMAD3**	BRCA1	1	19
**LIMS1**	ASPM	1	4
**ASH2L**	BRCA1	1	2
**PRDX3**	LRRK2	1	1
**EIF6**	ASPM	1	4
**AKT1**	BRCA1	1	6
**CDC16**	ASPM	1	4
**UBE2E3**	BRCA1	1	1
**MED17**	BRCA1	1	4
**CTSD**	APP	1	1
**SSFA2**	BRCA1	1	1
**CALU**	ASPM	1	8
**SP1**	BRCA1	1	27
**ITSN2**	PSEN1	1	1
**LOX**	APP/PSEN1	3	6
**MAPK8IP1**	APP	1	3
**FHL2**	BRCA1/APP	1	6
**MED1**	BRCA1	1	6
**STAMBPL1**	PSEN1	1	5
**ARRB1**	APP	1	7
**PDS5B**	BRCA1	2	5
**MFAP5**	APP/PSEN1	3	2

**SPRY2**	APP/PSEN1	3	1
**CLSTN1**	APP/PSEN1	1	4
**MID2**	ASPM	1	3
**RPL6**	DMD	1	16
**PRAM1**	APP/PSEN1	1	4
**FBXL12**	PSEN1	1	7
**SNCA**	APP/LRRK2	1	7
**FANCI**	BRCA1	1	9
**SPPL2B**	PSEN1	1	2
**PPP6R2**	ASPM	1	4
**BRCC3**	BRCA1	1	11
**DOCK3**	PSEN1	1	3
**LPAR6**	DMD	1	1
**NUP153**	BRCA1	1	5
**PRKAA1**	ASPM	1	5
**NCOA2**	BRCA1	1	6
**RNF115**	APP	1	3
**PIK3R1**	PSEN1	1	11
**MAP2K7**	LRRK2	1	1
**EGLN3**	ASPM/BRCA1	1	42
**APP**	APP	self	109
**TNS3**	DMD	1	8
**BCCIP**	ASPM/BRCA1	2	5
**PITRM1**	APP	1	1
**SDCCAG3**	ASPM	1	5
**FANCD2**	BRCA1	1	16

**KIF11**	BRCA1/RBM45	1	4
**RELA**	BRCA1	1	9
**SNX7**	ASPM	1	2
**BARD1**	BRCA1	1	20
**VPS28**	BRCA1/DMD	2	2
**PSEN2**	APP/PSEN1	1	59
**LTBP1**	PSEN1	2	6
**FN1**	PSEN1	2	9
**RBBP7**	BRCA1	1	6
**RPA1**	BRCA1	1	25
**BGN**	APP	1	1
**FBN1**	APP/PSEN1	2	20
**LRP2**	APP	1	4
**CLSPN**	BRCA1	1	2
**CRTAC1**	ASPM	1	1
**FBLN2**	APP/PSEN1	3	4
**AURKA**	BRCA1	1	17
**CDK2**	BRCA1	1	30
**EIF5B**	BRCA1	1	1
**APBB1**	APP	1	3
**ZNF408**	APP/PSEN1	3	5
**RNF32**	APP/PSEN1	1	20
**CTNNBL1**	RBM45	1	3
**TUBGCP5**	ASPM	1	5
**APH1A**	PSEN1	1	7
**APBA1**	APP/PSEN1	1	5

**CDK16**	BRCA1	1	1
**ABL1**	BRCA1	1	14
**CALR**	APP	1	9
**SUMO1**	BRCA1	1	13
**TMCC2**	APP	1	1
**DAB1**	APP	1	2
**ACHE**	APP	1	1
**MAPK8IP2**	APP	1	3
**MAP3K3**	BRCA1	1	1
**CDH8**	PSEN1	1	6
**CLK1**	APP/PSEN1	2	2
**PROX1**	APP/PSEN1	3	1
**EZH2**	BRCA1	1	6
**PRDX2**	APP/PSEN1	1	10
**SLC25A10**	ASPM	1	3
**MSH3**	BRCA1	1	4
**UBQLN1**	APP/PSEN1	1	9
**SDF4**	ASPM/BRCA1	2	3
**ANKRD28**	BRCA1	1	1
**LONRF3**	DMD	1	1
**TP63**	ASPM	1	4
**VIM**	LRRK2	1	2
**SNTA1**	DMD	1	11
**COPS7B**	ASPM	1	3
**MAP2**	APP	1	1
**NELFB**	BRCA1	1	3

**MGEA5**	APP/PSEN1	2	5
**CDS1**	BRCA1	1	1
**HNF4A**	DMD	1	2
**TGFBR1**	PSEN1	1	7
**YWHAQ**	LRRK2	1	7
**NSMCE4A**	BRCA1	1	3
**PACS2**	ASPM	1	1
**RBMX**	RBM45	1	9
**DNAJA1**	BRCA1/LRRK2	1	4
**AURKB**	ASPM/BRCA1/DMD/LRRK2	2	8
**JUP**	PSEN1	1	16
**UBE2D1**	BRCA1	1	2
**DNER**	PSEN1	1	2
**EP300**	BRCA1	1	18
**PSMD6**	BRCA1	1	8
**RAD23A**	PSEN1	1	11
**KDM1A**	BRCA1	1	7
**AFG3L2**	RBM45	1	2
**CDC37**	APP/PSEN1/LRRK2	1	26
**YWHAH**	LRRK2	1	7
**PLEKHA7**	PSEN1/BRCA1/DMD/APP/LRRK2	2	10
**NFATC2**	APP/PSEN1	3	3
**ERGIC3**	APP/PSEN1	2	1
**PRRC2B**	APP/PSEN1	3	1
**GDI1**	APP/PSEN1	2	2
**USP4**	APP/PSEN1	2	3

**WBP4**	RBM45	1	2
**MEMO1**	RBM45	1	2
**FASLG**	DMD	1	1
**LOXL4**	LRRK2/APP/PSEN1	2	5
**KRT8**	BRCA1/DMD	2	1
**IRAK2**	PSEN1	1	3
**CSNK2A1**	APP/BRCA1	1	20
**LRP8**	APP	2	1
**POLN**	BRCA1	1	9
**BACH1**	BRCA1	1	9
**PSMA5**	PSEN1/APP	1	6
**TERF2**	DMD	1	4
**APBA2**	APP	1	4
**PSEN1**	PSEN1	self	100
**IRAK1**	APP/PSEN1	2	6
**LMNA**	BRCA1	1	18
**BRAP**	BRCA1	1	1
**NFYA**	BRCA1	1	4
**PUM2**	RBM45	1	4
**TUBG1**	BRCA1	1	6
**GAPDH**	APP/PSEN1	1	11
**STUB1**	ASPM/LRRK2	1	15
**HPS1**	APP/PSEN1	2	1
**DISC1**	DMD/APP	2	2
**NOTCH2**	PSEN1	1	7
**SH3GL2**	LRRK2	1	1

**RPUSD4**	RBM45	1	2
**SGK1**	DMD	1	4
**DYNC1H1**	APP/PSEN1	2	3
**CFL1**	APP/PSEN1	1	6
**MYL9**	LRRK2	1	1
**BAG5**	LRRK2	1	3
**HDAC1**	BRCA1/DMD	1	29
**SNTB2**	BRCA1/DMD/PSEN1	2	9
**YWHAB**	LRRK2	1	8
**FBLN5**	APP/PSEN1	3	5
**NCSTN**	APP/PSEN1	1	18
**NPLOC4**	APP/PSEN1	2	3
**MYO1B**	LRRK2	1	1
**KIF23**	DMD	1	2
**HSD17B10**	APP	1	1
**NUMB**	APP	1	1
**STAU1**	APP	1	1
**CTNNA1**	APP/DMD/PSEN1	1	13
**KCNJ12**	DMD/PSEN1	2	3
**CASP7**	PSEN1	1	9
**SCARB1**	DMD/APP/PSEN1	3	2
**KCNIP4**	APP/PSEN1	1	3
**MAPT**	APP/LRRK2/PSEN1	1	21
**RPS20**	LRRK2	1	2
**MAPK6**	ASPM	1	21
**RPL31**	BRCA1	1	1

**HERPUD1**	APP/PSEN1	1	4
**JAK2**	BRCA1	1	2
**KIF15**	DMD	1	1
**CUL3**	ASPM/RBM45	1	26
**FYN**	BRCA1/DMD	2	5
**MYL2**	ASPM	1	1
**FANCL**	BRCA1/RBM45	1	2
**SRI**	APP/PSEN1	2	1
**MCPH1**	BRCA1	1	6
**ATRIP**	BRCA1	1	3
**MPP6**	DMD	1	3
**HTRA2**	APP/PSEN1	1	3
**NMI**	BRCA1	1	4
**MTOR**	ASPM	1	3
**CASP3**	APP/PSEN1	1	16
**TRAF2**	APP/PSEN1	2	6
**IRF4**	APP/PSEN1	2	23
**PPP2CA**	BRCA1	1	11
**RBM45**	RBM45	self	37
**PALB2**	BRCA1	1	14
**SLC40A1**	APP	1	1
**HSP90B1**	ASPM	1	10
**TNS2**	DMD/PSEN1	2	3
**RB1**	BRCA1	1	24
**XRCC5**	BRCA1	1	3
**KIF2A**	ASPM	1	1

**MXD3**	ASPM	1	1
**CHKA**	APP	1	2
**EAF1**	ASPM	1	2
**VCAM1**	PSEN1/BRCA1/DMD/APP/LRRK2	2	15
**LRRK2**	LRRK2	self	49
**MSN**	LRRK2	1	1
**ACTN1**	PSEN1	1	4
**ECD**	BRCA1/APP/PSEN1	2	2
**STAT1**	BRCA1	1	6
**EFEMP2**	APP/PSEN1	3	4
**CSNK1D**	APP	1	5
**UBE2W**	BRCA1	1	1
**MARK2**	DMD/PSEN1	2	4
**HDAC2**	BRCA1	1	14
**BRIP1**	BRCA1	1	3
**CFLAR**	APP/PSEN1	2	6
**CTNNB1**	APP/PSEN1	1	52
**FARSA**	APP/PSEN1	2	3
**RBBP4**	BRCA1	1	8
**CCNE1**	BRCA1	1	4
**MYOC**	APP/PSEN1	3	2
**SNAPIN**	LRRK2	1	1
**TRIM41**	APP	1	1
**PPP1CA**	BRCA1	1	6
**NCAPH2**	ASPM	1	6
**ESR1**	BRCA1	1	18

**DSN1**	ASPM/BRCA1	2	5
**DTNBP1**	ASPM/BRCA1/DMD	2	3
**YWHAZ**	APP/LRRK2	1	24
**TGFB1**	APP	1	2
**KIFC1**	ASPM	1	2
**UIMC1**	BRCA1	1	6
**RAD51**	BRCA1	1	29
**PRKACA**	LRRK2	1	10
**RMDN3**	PSEN1	1	1
**LRP6**	LRRK2	1	1
**TARS**	BRCA1	1	1
**AP1M2**	APP/PSEN1	1	4
**NUMA1**	ASPM	1	6
**RPL23**	LRRK2	1	1
**RBBP8**	BRCA1	1	6
**MAP2K3**	LRRK2	1	2
**NAP1L1**	ASPM	1	8
**EXOC6**	APP	1	6
**ACTG1**	LRRK2	1	9
**RPS15**	LRRK2	1	1
**GPN3**	ASPM/BRCA1	2	1
**HGS**	APP	1	3
**DNAJB1**	APP	1	5
**XPO1**	BRCA1/DMD	1	33
**DLL1**	PSEN1	1	3
**INTU**	ASPM	1	2

**MIER2**	ASPM/BRCA1	2	1
**HNRNPD**	BRCA1	1	3
**PGM5**	DMD	1	2
**FANCG**	BRCA1	1	8
**SMARCA4**	BRCA1	1	11
**MAP1B**	LRRK2	1	1
**ETFA**	PSEN1	1	2
**TXN2**	RBM45	1	1
**PABPC1**	BRCA1	1	2
**CSNK1A1**	PSEN1/APP/LRRK2	2	6
**IL1R1**	PSEN1	1	5
**KLC1**	APP	1	1
**RBM42**	RBM45	1	4
**DMC1**	BRCA1	2	2
**YME1L1**	PSEN1	1	3
**CASP6**	APP/PSEN1	1	10
**CASP8**	APP/PSEN1	1	15
**EMSY**	BRCA1	1	3
**MFAP2**	APP/PSEN1	3	3
**DSP**	PSEN1	1	6
**UBE2N**	BRCA1	1	4
**UBE2I**	BRCA1	1	9
**TLK2**	APP/PSEN1	3	2
**NOS1**	DMD	1	2
**LMO4**	BRCA1	1	2
**PCMT1**	APP/PSEN1	2	2

**EPN2**	BRCA1/APP/LRRK2	3	1
**CTNND2**	PSEN1	1	6
**CCDC83**	ASPM	1	1
**MLF1**	BRCA1	2	4
**TP53INP1**	ASPM	1	2
**EFNB2**	PSEN1	1	2
**RHEB**	PSEN1	1	4
**CLTC**	BRCA1/APP/LRRK2	2	7
**ATM**	BRCA1	1	17
**APBB2**	APP	1	2
**UBA1**	BRCA1	1	4
**CDC45**	ASPM/BRCA1	2	6
**TCF7L2**	PSEN1	1	3
**CNKSR1**	ASPM	1	1
**RCN2**	ASPM	1	7
**JAK1**	BRCA1	1	2
**EEF1G**	DMD/APP	2	2
**MDC1**	BRCA1	1	21

Table S3 – Description of Primary Vertebrate Proteins Used in Non-Interacting Pairs

Primary protein	Number of pairings
**APOE**	22
**ASPM**	257
**DMD**	230
**FBN1**	267
**IRF4**	285
**LRRK2**	271
**NLRP3**	87
**PSEN1**	275
**RBM45**	283

Table S4 – Alzheimer's disease Analysis Information by Protein

Alzheimer's disease associated protein	Number of orthologs	Number of paired proteins with sufficient orthologs	Used in Analysis	Number of pairs with interaction chances above 90%	Number of pairs with interaction chances above 50%
**ABCA7**	117	621	Yes	7	62
**ABI3**	167	0	No	0	0
**ADAM10**	236	1451	Yes	129	814
**ADAMTS1**	245	3029	Yes	50	517
**ADAMTS4**	146	0	No	0	0
**ALPK2**	221	6510	Yes	25	258
**APH1B**	121	4	Yes	0	0
**APOC1**	108	0	No	0	0
**APOE**	133	4	Yes	0	1
**APP**	235	1353	Yes	269	1093
**BCKDK**	206	0	No	0	0
**BIN1**	209	224	Yes	13	87
**BZRAP1**	<100	0	No	0	0
**CASS4**	262	2711	Yes	34	371
**CCL2**	<100	0	No	0	0
**CD2AP**	240	550	Yes	25	207
**CD33**	<100	0	No	0	0

**CELF1**	261	844	Yes	194	710
**CLNK**	207	7	Yes	0	0
**CLU**	217	84	Yes	0	13
**CNN2**	193	8	Yes	0	0
**CNTNAP2**	201	1707	Yes	94	714
**CR1**	<100	0	No	0	0
**CSTF1**	283	822	Yes	268	755
**CYB561**	<100	0	No	0	0
**ECHDC3**	232	0	No	0	0
**EED**	264	45	Yes	11	36
**EPHA1**	192	1861	Yes	82	436
**FERMT2**	243	1916	Yes	347	1376
**GPR141**	170	3	Yes	0	0
**HESX1**	241	0	No	0	0
**HLA-DQA1**	<100	0	No	0	0
**HLA-DRB1**	<100	0	No	0	0
**IL34**	231	0	No	0	0
**INPP5D**	244	2190	Yes	49	448
**IQCK**	241	0	No	0	0
**KAT8**	195	0	No	0	0
**MEF2C**	270	620	Yes	201	512
**MS4A4E**	<100	0	No	0	0
**MS4A6A**	<100	0	No	0	0
**NYAP1**	147	95	Yes	24	66
**OARD1**	264	0	No	0	0
**PICALM**	254	1233	Yes	278	1039
**PILRA**	<100	0	No	0	0
**PLCG2**	246	4696	Yes	144	842
**PLD3**	203	50	Yes	6	21
**PSEN1**	213	32	Yes	1	11
**PSEN2**	233	117	Yes	8	46
**PSMC3**	276	698	Yes	181	636
**PVRL2**	<100	0	No	0	0
**RAB10**	252	0	No	0	0
**RP11-81K2.1**	<100	0	No	0	0
**SCIMP**	124	0	No	0	0
**SLC24A4**	227	5	Yes	0	0
**SORL1**	242	8360	Yes	83	809
**SPI1**	259	16	Yes	0	6
**SPPL2A**	241	693	Yes	29	247
**TREM2**	120	6	Yes	0	5
**UNC5CL**	123	0	No	0	0
**USP6NL**	241	1676	Yes	37	360

**WWOX**	243	62	Yes	6	32
**ZCWPW1**	126	1	Yes	0	1

Table S4: *A summary of the genes used in the Alzheimer's disease analysis including whether they were annotated in RefSeq, how many species had orthologs annotated in RefSeq, how many other proteins had orthologous genes spanning the same 100 species as the target protein (i.e., paired orthologs), and whether the protein was included in the analysis. The number of predicted protein interactions at 90% and 50% thresholds for each protein are shown.*

Table S5 – Potential protein interactions with Alzheimer's disease-associated proteins

**Potential protein interactions with Alzheimer's disease-associated proteins**												
**Score threshold**	**480.76**	**151.52**	**79.29**	**30.18**	**45.61**	**20.99**	**15.04**	**10.93**	**7.85**	**6.17**	**4.62**	**3.59**
**Percent chance of interaction**	**99.68**	**98.43**	**97.32**	**95.85**	**92.23**	**89.40**	**85.31**	**80.93**	**75.51**	**69.99**	**63.81**	**55.96**
**Number of interactions**	262	449	496	726	765	972	1184	1441	1831	1496	1928	1887
**Percent previously documented**	0.77	1.81	1.43	1.26	0.79	0.83	0.34	0.42	0.44	0.07	0.47	0.21

Table S5: *A breakdown of the scores from the Alzheimer's disease analysis that demonstrated a chance of interaction.*
